# Special Care Dentistry and COVID-19 Outbreak: What Lesson Should We Learn?

**DOI:** 10.3390/dj8020046

**Published:** 2020-05-09

**Authors:** Arkadiusz Dziedzic

**Affiliations:** Department of Conservative Dentistry with Endodontics, School of Medicine with the Division of Dentistry in Zabrze, SUM, Medical University of Silesia in Katowice, Pl. Akademicki 17, 41-902 Bytom, Poland; adziedzic@sum.edu.pl

**Keywords:** special care dentistry, COVID-19, coronavirus SARS-CoV-2, service provision

## Abstract

The recent outbreak of coronavirus disease 2019 (COVID-19) caused by the emerging severe acute respiratory syndrome coronavirus 2 (SARS-CoV-2) and the declaration of pandemic by the World Health Organization have made an enormous impact on medical and dental care across the world. The current COVID-19 situation may teach dental teams a better approach and optimal ways concerning the management of patients with special needs, by bringing people together to discuss and optimize standards of care, as often happens in challenging situations. We can always learn new things that turn out to be valuable and useful even in exceptionally difficult times, and in addition, dental services can benefit from enabling positive attitudes and introducing constructive changes. Clinicians just need to keep in mind that adjustment to a new future reality appears inevitable for both patients and professionals who provide care.

The COVID-19 pandemic is undoubtedly posing the greatest challenge that special care dental services ever had to face, but we can learn from this situation and benefit from this challenge. In fact, we should realize that this is an opportunity for constructive change, toward becoming more patient-centered and, more importantly, more adaptive providers, equipped with a strategy for positive change [1]. It is the right time to re-define and re-think our role, trying to learn the lesson that the current situation is teaching us and prepare well for the future, by changing our mindset and widening our resources. The “3R” principle, (R)edefinition, (R)econsideration and (R)eflections, addresses well these challenges in the dental sector, recommending clinicians to demonstrate a professional commitment and be ‘slicker and quicker’, displaying critical decision-making skills. 

One of the positive aspects of the current COVID-19 outbreak is the fact that dental teams all over the world and their patients have instantly understood in a profound way the important role of primary, community, and hospital services [2], which continually contribute, despite some interruption, to the maintenance of patients’ wellbeing. Without the essential urgent and emergency dental care provided by dental services, a large number of patients would be deprived of appropriate help [3]. Nowadays, we might clearly realize that dental care constitutes a crucial element in the whole healthcare system and public health maintenance, and, more importantly, that basic ‘impactful dental interventions’ are critical for pain management in general [4]. Despite the fact that relevant key expressions such as triaging, prioritizing, compromising, and making difficult choices have become a daily reality in this pandemic time, these actions can bring an ‘added value’ and improve our service. We can definitely enhance our communication skills for tele(video) consultations, providing comprehensive advice to patients in needs. In addition, and more importantly, we have a great opportunity to create a unique, professional relationship with our patients, based on our commitment and profound understanding of clinical problems. As the impact of COVID-19 on wellbeing and mental health appears to be significant, dental services will need to be able to provide some sort of ‘psychological counselling and reassurance’ prior to dental care to vulnerable individuals with complex pre-existing conditions and special requirements. This is why improving our skills is essential. 

From the pragmatic and clinical perspective, questions are arising on a daily basis; for instance, how can we help our phobic patients when most of sedation and dental general anesthesia services are currently suspended and these patients are known not to tolerate any form of standard local anesthesia? A broader implementation of non-pharmacological pain and anxiety control measures would be inevitable and the only available option [5]. This is definitely the proper time to expand our panel of treatment and/or ‘psychological’ methods and learn/revise in depth alternative techniques, regardless of patient’s age, e.g., gradual exposure, hypnotherapy, behavioral management, professional cognitive behavioral therapy (CBT), the use of virtual goggles for distraction, desensitization methods, etc. [6,7,8]. This would be particularly useful when treating individuals with learning disabilities, young patients with attention deficit hyperactivity disorder (ADHD), phobic children. Nowadays, we also need to change our standard way of dealing with severe cases of medically compromised patients, trying to balance and weight our clinical decisions and reviewing service capacity and patient’s safety on a regular basis. Therefore, we must act as effectively as we can, trying to ‘alter our mindset’ by adapting it to the new reality and setting up far more adjustable dental services. Undoubtedly, the recently implemented strict cross infection control measures and the awareness of infectious diseases transmission have affected dental services management in a positive way, as they are leading to a better level of infection prevention control and better personal protective measures. 

In the field of special care dentistry, even basic advice may have a huge impact on clinical outcome. A patient with acute/chronic oral medicine problems would vastly benefit from professional recommendations, provided over the phone, regarding treatment planning [9]. After all, it is vitally important to reassure our patients, encouraging them to continue taking their prescribed medications, as any sudden change in pharmacotherapy regimen may have a detrimental effect on patients’ outcome [10]. We must encourage our patients to continue to access health systems, particularly in case of emergency problems, and ensure that dental care is able to support them. This is a primary role of reorganized dental services to minimize an indirect impact of COVID-19 on oral health; therefore, preparation seems to be another key word for special dental care ‘evolution’ in the nearest future.

The overall impact of SARS-CoV-2 on general health is deemed to be far more profound, with significant effects on various systems and functions, including the respiratory and cardiovascular systems, hemostasis, cognitive and renal functions [11,12,13,14,15]. When the pandemic crisis is finally over, special care dentistry teams should be well prepared to tackle increased numbers of patients with unresolved medical and dental problems or unfinished treatment courses, as a consequence of deferred services. For some of them, restorative care will no longer be an option, and more radical planning will be necessary. Consequently, we should expect oral health deterioration and increased incidence of oral diseases. Moreover, patients who were treated in intensive care units due to COVID-19 serious complications may require special attention as a group at high risk for oral health deterioration [16]. 

Nevertheless, the lesson we all need to learn is simple: be adaptable, be empathetic, and always try to provide the best care to your patients, while learning from challenges. 
